# PAVING THE WAY FORWARD IN HOUSING WITH SERVICES: THE RIGHT CARE, RIGHT PLACE, RIGHT TIME PROGRAM

**DOI:** 10.1093/geroni/igz038.2851

**Published:** 2019-11-08

**Authors:** Edward A Miller, Pamela Nadash, Alisha Sanders

**Affiliations:** 1 University of Massachusetts Boston, Boston, Massachusetts, United States; 2 LeadingAge, Washington, D.C., United States

## Abstract

Older people living in congregate environments are obvious beneficiaries of supportive services. The potential for prevention is clear, particularly among low-income elders living in subsidized housing; it is this group that is at high risk for significant healthcare and other costs, and it is this group that suffers considerably from a fragmented healthcare system. Policymakers have long seen the advantages of reaching this population, but most existing housing with services programs have focused more on social than health-related supports. The Right Care, Right Place, Right Time initiative (R3) was launched in July 2017 to demonstrate the value of supportive services to seniors living independently in affordable housing in the Greater Boston area, while reducing health care costs. The R3 program consists of two on-site wellness teams, including a wellness nurse and wellness coordinator. Each team is responsible for about 200 participants across two housing sites. The R3 evaluation included both quantitative and qualitative components. The quantitative component entails pre/post comparison as well as a control group analysis, focusing on various health and health utilization outcomes. The qualitative component includes key informant interviews examining program development and implementation and focus groups capturing the resident experience. The purpose of this symposium is for evaluation team members to report on the experiences of program participants, administrators/staff, housing managers/staff, and community partners with the R3 program, and to assess program impact. Edward Miller and Pamela Nadash will serve as chair and co-chair, respectively; Alisha Sanders as the discussant.

